# Increased hippocampal epigenetic age in the Ts65Dn mouse model of Down Syndrome

**DOI:** 10.3389/fnagi.2024.1401109

**Published:** 2024-05-21

**Authors:** Francesco Ravaioli, Fiorenza Stagni, Sandra Guidi, Chiara Pirazzini, Paolo Garagnani, Alessandro Silvani, Giovanna Zoccoli, Renata Bartesaghi, Maria Giulia Bacalini

**Affiliations:** ^1^IRCCS Istituto delle Scienze Neurologiche di Bologna, Bologna, Italy; ^2^Department for Life Quality Studies, University of Bologna, Rimini, Italy; ^3^Department of Biomedical and Neuromotor Sciences, University of Bologna, Bologna, Italy; ^4^Department of Medical and Surgical Sciences (DIMEC), University of Bologna, Bologna, Italy; ^5^IRCCS Azienda Ospedaliero-Universitaria di Bologna, Bologna, Italy; ^6^PRISM Lab, Department of Biomedical and Neuromotor Sciences, University of Bologna, Bologna, Italy

**Keywords:** down syndrome, epigenetic clock, Ts65Dn, aging, hippocampus, DNA methylation

## Abstract

Down syndrome (DS) is a segmental progeroid genetic disorder associated with multi-systemic precocious aging phenotypes, which are particularly evident in the immune and nervous systems. Accordingly, people with DS show an increased biological age as measured by epigenetic clocks. The Ts65Dn trisomic mouse, which harbors extra-numerary copies of chromosome 21 (Hsa21)-syntenic regions, was shown to recapitulate several progeroid features of DS, but no biomarkers of age have been applied to it so far. In this pilot study, we used a mouse-specific epigenetic clock to measure the epigenetic age of hippocampi from Ts65Dn and euploid mice at 20 weeks. Ts65Dn mice showed an increased epigenetic age in comparison with controls, and the observed changes in DNA methylation partially recapitulated those observed in hippocampi from people with DS. Collectively, our results support the use of the Ts65Dn model to decipher the molecular mechanisms underlying the progeroid DS phenotypes.

## 1 Introduction

Down syndrome (DS) is a common genetic disorder caused by complete or segmental triplication of chromosome 21 (Hsa21) and is the most frequent genetic cause of intellectual disability. DS is considered a segmental progeroid syndrome, characterized by a precocious aging-like deterioration that is particularly evident at the immune system and brain level. This view, originally proposed by George Martin based on the analysis of DS phenotypic traits (Martin, 1978), has been further refined in the last two decades through physiological and molecular analyses that explored similarities and differences between the pillars of aging and alterations occurring in DS (Zigman, 2013; Franceschi et al., 2019; Chen et al., 2021). In this framework, several biomarkers of age have been explored in people with DS, including those based on telomere length, magnetic resonance neuroimaging (brain age), serum protein glycosylation (GlycoAge), and DNA methylation (DNAm) (epigenetic clocks) [reviewed in Franceschi et al. (2019)]. These studies concordantly suggest that people with DS are biologically older than their chronological age.

Murine models are largely used in the study of aging and age-related diseases (Palliyaguru et al., 2021), and mouse epigenetic biomarkers of age have been developed (Stubbs et al., 2017; Coninx et al., 2020; Zhou et al., 2022). So far, however, these epigenetic clocks have been applied to a limited extent, and, to the best of our knowledge, no data are available for mouse models of DS.

The Ts65Dn mouse strain is the most common model for the study of DS. These mice are segmentally trisomic for a region of chromosome 16 that is homologous to part of Hsa21. Ts65Dn mice were shown to recapitulate a wide range of DS-specific behavioral, physiological, and neuroanatomical features such as reduced brain size, neuronal density (Stagni et al., 2018), altered neuronal function (Kleschevnikov et al., 2004), and altered dendrite architecture in hippocampal regions (Uguagliati et al., 2022), as well as spatial learning and memory deficits. In addition, the Ts65Dn mouse shares with human DS a multi-systemic premature aging condition associated with early alterations in mitochondrial functions, DNA damage response and early neurodegeneration. (Mollo et al., 2020; Kirstein et al., 2022; Puente-Bedia et al., 2022).

In this pilot study, we aimed to evaluate hippocampal epigenetic age in the Ts65Dn model. We used a hippocampus-specific mouse epigenetic clock developed by Zymo Research (referred to as DNAge®), which is based on deep bisulfite sequencing of 300 target regions containing 2045 CpG sites. Using this clock, Coninx et al. previously reported an increase in epigenetic age in the triple transgenic Alzheimer’s disease mouse model (Coninx et al., 2020).

## 2 Materials and methods

### 2.1 Sample collection

Ts65Dn and euploid mice were generated by crossing B6EiC3Sn a/A-Ts(17^16) 65Dn females (JAX line 1924) with C57BL/6JEiJ × C3H/HeSnJ (B6EiC3Sn) F1 hybrid males (JAX line 1875; euploid males) that were supplied by Jackson Laboratories (Bar Harbor, ME, United States). Ts65Dn (5 males and 1 female) and euploid (6 males and 1 female) littermates aged 20 weeks were anesthetized, and the hippocampi were quickly collected and immediately snap-frozen in liquid nitrogen. DNA was isolated from the right hippocampal region using AllPrep DNA/RNA/miRNA Universal Kit (QIAGEN, Hilden, Germany).

### 2.2 DNAm preprocessing and DNAge® prediction

DNA samples were stabilized using DNA/RNA Shield reagent (1:3 ratio) and shipped to the service provider (Zymo Research) for downstream processing. The samples were processed in two separate, group-balanced batches. DNA bisulfite conversion, library preparation, sequencing, DNAm values extraction, and epigenetic age prediction with DNAge® clock were performed by Zymo Research (Orange, CA, United States) as previously reported (Coninx et al., 2020). Briefly, genomic DNA recovered from the DNA/RNA shield solution was bisulfite-converted using an EZ DNAm-Lightning Kit (Zymo Research, Irvine, CA). Sequencing libraries were prepared according to the Simplified Whole-panel Amplification Reaction Method (SWARM®). Sequencing was run on an Illumina NovaSeq platform for at least 1,000X sequencing depth per CpG position. Sequences were identified using Illumina base-caller software and then aligned to the reference genome (GRCm38/mm10) using Bismark (Krueger and Andrews, 2011). Methylation levels for each assayed cytosine were calculated as the ratio between the number of reads reporting a C and the number of reads reporting either a C or a T (beta value). DNAm *beta* values of CpG sites making up the DNAge® epigenetic clock (*n* = 2040) were obtained from the Zymo Research sequencing service. The DNAge® epigenetic clock comprises 300 loci, ranging from 1 bp to 444 bp in length, harboring from 1 to 34 CpG dinucleotides. In each locus, adjacent CpGs are less than 150 bp apart.

### 2.3 Statistical analysis

All analyses were performed in R (v 4.2.3). Differences in DNAge® epigenetic age between Ts65Dn and euploid mice were evaluated using the Mann–Whitney test. DNAge® epigenetic age variance between Ts65Dn and euploid mice was analyzed using the F-test. The Mann–Whitney test was used to assess DNAm differences between Ts65Dn and euploid mice at a single CpG level. Similarly, the Mann–Whitney test was used to assess DNAm differences between Ts65Dn and euploid male mice at a single CpG level. The Benjamini–Hochberg (BH) method was used for multiple test correction. CpGs with a Mann–Whitney *p*-value < of 0.0000245 (corresponding to a BH-adjusted *p*-value <0.05) were considered significant after FDR correction, while CpGs with a Mann–Whitney *p*-value of <0.01 were considered nominally significant. The loci targeted by the DNAge® clock that contained at least two nominally significant (Mann–Whitney *p*-value<0.01) CpG sites were referred to as differentially methylated regions (DMRs).

### 2.4 Integration with Hsa21 human hippocampal DNAm data

DNAm data from Hsa21 human hippocampus samples were downloaded from Gene Expression Omnibus (GEO) repositories GSE63347, GSE129428, and GSE64509. To convert genomic coordinates of DNAge® epigenetic clock CpGs from mouse (mm10) to human (hg19), the *liftOver* UCSC tool was used. The coordinates of 86% (*n* = 1747) of DNAge® CpGs were successfully lifted over from mm10 to hg19. Only 14 of the hg19-converted murine CpG sites exactly overlapped with a microarray probe, which is expected on the basis of the limited genomic coverage of the Illumina Infinium 450 k platform. As it is well established that DNAm values of genomically adjacent CpG sites tend to be correlated, we also included in our analysis the microarray probes flanking the lifted-over CpG site. In particular, we considered the 250 bp upstream and the 250 bp downstream of the hg19-converted murine CpG site, which resulted in 440 Illumina Infinium 450 k probes. The GSE63347 dataset contains Infinium 450 K DNAm data from the hippocampi of two subjects with DS (age: 42, 57 years, two men) and seven euploid controls (age: 38–64, two men and five women). DNAm *beta* values were compared between DS and euploid controls using the Mann–Whitney test. The GSE129428 and GSE64509 datasets contain Illumina Infinium 450 K DNAm data from 25 hippocampi (age range: 34–78 years) and 32 hippocampi (age range: 38–114 years), respectively, obtained from subjects without any overt pathology. The association with age was calculated by fitting a linear model to each CpG probe (converted to M values as above). Correction for multiple testing was performed using the BH method.

## 3 Results

**Figure 1:**
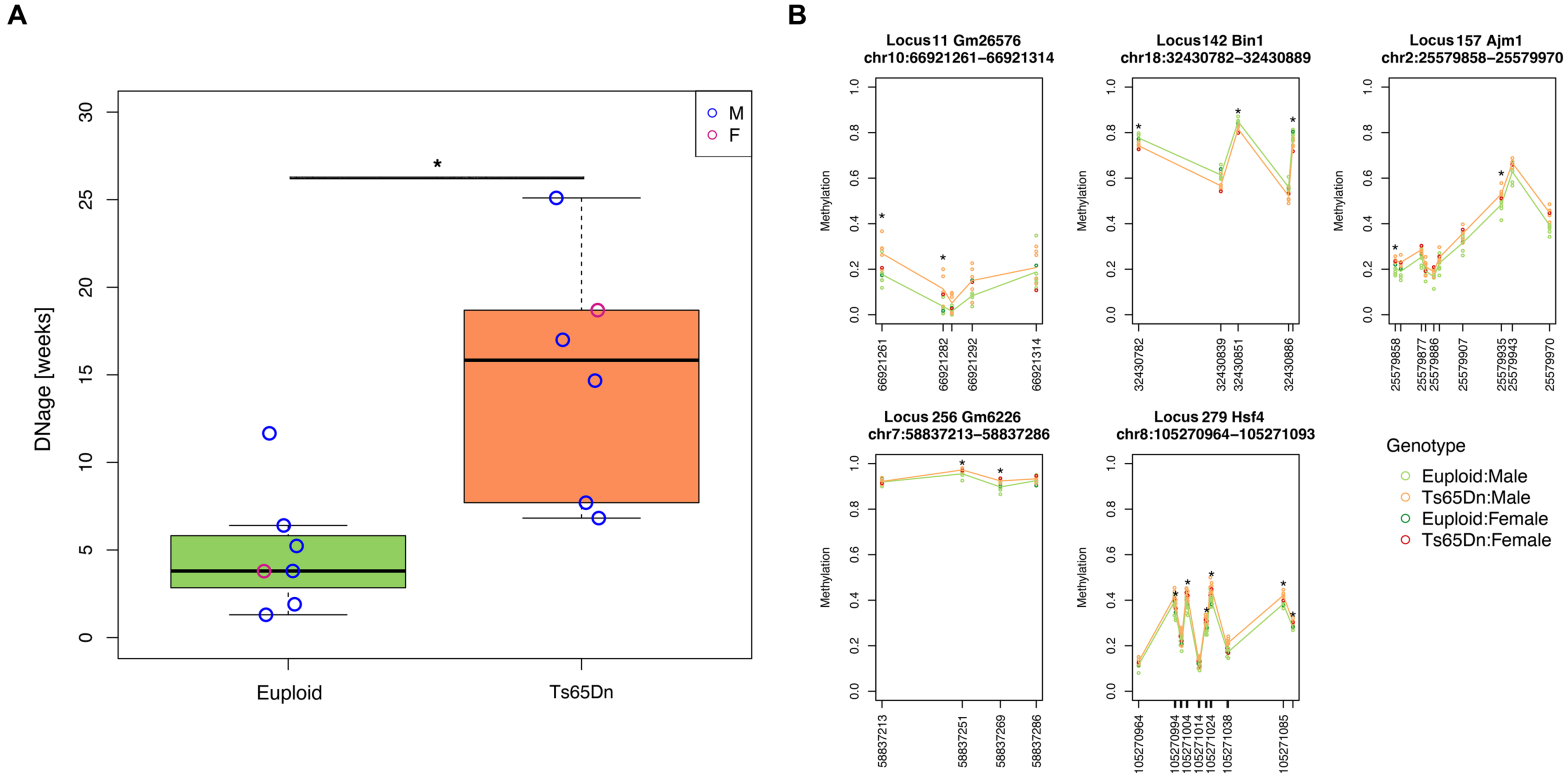
Increased epigenetic age of hippocampi in Ts65Dn mice. **(A)** The boxplot shows the epigenetic age of the hippocampus of Ts65Dn and euploid mice (including both male and female animals) predicted using the Zymo Research DNAge® algorithm. The blue and red circles indicate data from male and female mice, respectively. *: nominal *p* < 0.01, Mann–Whitney test. **(B)** The line plots of average DNAm profiles of Ts65Dn (orange line) and euploid mice (green line) (including both male and female animals) for differentially methylated regions with at least two significant CpG sites. *: nominal *p* < 0.01, Mann–Whitney test. The dark green and red circles indicate data from euploid and Ts65Dn female mice, respectively. The light green and orange circles indicate data from euploid and Ts65Dn male mice, respectively.

We applied the DNAge® clock to assess the hippocampal epigenetic age in seven euploids (age: 20 weeks, six males and one female) and six Ts65Dn (age: 20 weeks; five males and one female) mice. The mean epigenetic age of euploid mice was 4.9 weeks, with a mean absolute deviation of 2.5 weeks. With respect to the estimated epigenetic age of euploid controls, Ts65Dn mice were significantly epigenetically older (mean epigenetic age:15 weeks; mean absolute deviation: 5.3 weeks; Mann–Whitney test *p*-value = 0.0047) (Figure 1A). The Ts65Dn female mouse was epigenetically older than the euploid female mouse (Figure 1A), and the epigenetic age differences between the two groups were marginally statistically significant when the analysis was restricted to male animals (Mann–Whitney test *p*-value = 0.017). To gain further insights into the DNAm differences that contribute to the increased epigenetic age of Ts65Dn mice, we tested DNAm differences at each CpG site comprising the DNAge® epigenetic clock. For each CpG site, Supplementary Table S1 reports the summary statistics, including those stratified by sex, and the results of the statistical analysis. No CpG site reached statistical significance after FDR correction. However, at the nominal level, we found 27 differentially methylated CpGs (nominal *p*-value<0.01), 21 of which were hypermethylated and 6 hypomethylated in Ts65Dn compared to euploid mice. Twelve of the 27 differentially methylated CpGs remained statistically significant even when considering only male mice, while the remaining 15 were marginally statistically significant (nominal *p*-value<0.05) (Supplementary Table S1). Furthermore, we identified five *loci* that contained at least two CpG sites with a nominal *p*-value<0.01 (Figure 1B). These differentially methylated regions (DMRs) are annotated to the *Bin1*, *Ajm1*, *Hsf4, Gm2662,* and *Gm26576* genes.

Finally, we performed a cross-species analysis to check whether the DNAge® CpG sites differentially methylated in Ts65Dn mice showed altered methylation in hippocampi from subjects with DS. We found a CpG probe (cg04235075) mapping within the *HSF4* gene that showed a trend toward hypermethylation in people with DS (GSE63347, nominal *p*-value = 0.05, not significant after FDR correction) (Supplementary Figure S1). For this CpG site, we also found a positive association with age in healthy human hippocampi. While in the GSE129428 dataset this association only reached nominal significance (nominal *p*-*value* = 0.039, not significant after FDR correction), possibly due to the narrow age range of samples, the same site showed a strong significant association with age in the GSE63347 dataset (*p*-value = 3e-04; FDR-corrected *p*-value = 0.011) (Supplementary Figure S1).

## 4 Discussion

In this pilot study, we reported for the first time that the epigenetic age of the hippocampus is higher in adult Ts65Dn mice compared to age- and sex-matched euploid controls.

We noticed that in our samples, the DNAge® epigenetic clock model that we used tended to underestimate the age of euploid mice (chronological age: 20 weeks; mean epigenetic age: 4.9 weeks). This finding is in line with the observations in the original publication (Coninx et al., 2020) and may be further modulated by the different strains used in our study. This effect may reflect the higher rate of neurogenesis in the adult hippocampus compared to other brain regions. Additionally, this effect seems to vary in different euploid mouse strains, although further evidence is required to confirm this observation. Despite this low accuracy, the DNAge® epigenetic clock showed good precision in euploid mice (mean absolute deviation: 2.5 weeks) and was not affected by batch effects, confirming its validity as a biomarker of age.

The observed increase in epigenetic age of the Ts65Dn mouse is fully in line with its progeroid phenotypes (see Introduction) and mimics the epigenetic aging previously described in the brain and blood of subjects with DS (Horvath et al., 2015; Mendioroz et al., 2015; Yu et al., 2020). At present, we cannot rule out that this increase is driven by potential confounding factors, including differences in the relative abundances of neuronal and non-neuronal cell types between the two mouse strains. In the Ts65Dn mouse hippocampus, in fact, neural progenitor cells exhibit a reduced proliferation rate, resulting in a reduction in the number of immature, proliferating, and mature neuronal cells (López-Hidalgo et al., 2016). This reduction mainly involves neurons but not astrocytes, the number of which may remain similar to that of euploid mice. In humans, brain epigenetic age estimated using the recently developed “Cortical clock” was inversely correlated with decreased neuronal cell proportions estimated from genome-wide DNAm data (Shireby et al., 2020), but this did not affect its association with neurodegenerative diseases such as Parkinson’s and Alzheimer’s diseases (Grodstein et al., 2021). As the epigenetic clock that we applied derives from a targeted assay, we are not able to estimate brain cell proportions, and further analysis at the cellular level is necessary to settle this issue.

Ts65Dn mice showed a higher DNAge variance compared to euploid mice, although they did not reach statistical significance (*F*-test *p*-value = 0.1231). This trend can be related to the progeroid phenotype, as an increase in epigenetic variability has been described during aging (Slieker et al., 2016), although we cannot exclude that it is the result of the phenotypic drift observed in the Ts65Dn model (Shaw et al., 2020).

An in-depth analysis of the sites making up the DNAge® epigenetic clock showed that some sites and regions showed a nominally significant association with trisomy. In particular, we identified five DMRs harboring CpGs nominally associated with trisomy. *Bin1* is a ubiquitously expressed gene that is known to modulate tau processing as well as to be involved in vesicle trafficking, inflammation, and apoptosis (Chapuis et al., 2013). *Ajm1* seems to be involved in cell-to-cell organization, while *Hsf4* is a transcription factor known to act upstream of several processes, including DNA damage repair (Cui et al., 2012). *Gm2662* and *Gm26576* functions are not known.

Finally, by comparing our findings in murine models of DS with available human hippocampal DNAm data, we showed that DNAm changes found for some CpG sites in Ts65Dn mice were concordant to those found for homologous genomic regions in people with DS and during physiological aging. Although these observations should be confirmed by larger samples and genome-wide studies, they suggest that the Ts65Dn model can mimic the epigenetic alterations and characteristics of DS.

A limitation of this study is related to the sex imbalance of the Ts65Dn and euploid cohorts, where only one female mouse is present per group. This hampers the possibility of properly assessing the effect of sex on epigenetic age and DNAm differences. To the best of our knowledge, no study has evaluated whether there are sex-related differences in the DNAge® epigenetic clock predictions, as it has been reported, for example, for Horvath’s human epigenetic clock (Horvath et al., 2016). In our cohort, the epigenetic ages of female mice were comparable to those of male animals in the corresponding group, suggesting that the observed differences between the Ts65Dn and euploid mice are not affected by sex. Similarly, the CpGs that were differentially methylated between trisomic and euploid mice were significant or marginally significant when restricting the statistical analysis to male animals. At the same time, the descriptive summary statistics reported in Supplementary Table S1 show that for several CpG sites, the DNAm values of female mice are outside the male DNAm intervals. Therefore, a rigorous assessment of sex-associated epigenetic age and DNAm differences should be performed in future studies.

In conclusion, our pilot study supports the use of the Ts65Dn model to decipher the molecular mechanisms underlying the progeroid DS phenotype. The increase in the hippocampal epigenetic age of DS mouse models and the DNAm differences at a single CpG level should be confirmed in a larger and more sex-balanced cohort. This cohort should also encompass other tissues and strains for a comprehensive assessment. In addition, our results support the use of the DNAge® clock and, possibly, other recently developed mouse epigenetic clocks (Zhou et al., 2022; Lu et al., 2023) as biomarkers not only of chronological but also of biological age. Such tools can be exploited to monitor the impact of disease-modifying interventions in DS.

## Data availability statement

The original contributions presented in the study are included in the article/Supplementary material, further inquiries can be directed to the corresponding author.

## Ethics statement

Ethical approval was not required for the study involving humans in accordance with the local legislation and institutional requirements. Written informed consent to participate in this study was not required from the participants or the participants' legal guardians/next of kin in accordance with the national legislation and the institutional requirements. The animal study was approved by Italian Ministry of Public Health, General Directorate of Animal Health and Veterinary Medicinal Products (456/2023-PR). The study was conducted in accordance with the local legislation and institutional requirements.

## Author contributions

FR: Data curation, Formal analysis, Investigation, Methodology, Writing – original draft, Writing – review & editing. FS: Conceptualization, Methodology, Resources, Supervision, Writing – review & editing. SG: Funding acquisition, Methodology, Resources, Writing – review & editing. CP: Writing – review & editing. PG: Writing – review & editing. AS: Writing – review & editing. GZ: Writing – review & editing. RB: Conceptualization, Writing – review & editing. MB: Conceptualization, Data curation, Formal analysis, Funding acquisition, Methodology, Supervision, Writing – original draft, Writing – review & editing.
