# Supplementary material for: Increased hippocampal epigenetic age in the Ts65Dn mouse model of Down Syndrome
Source: Front Aging Neurosci. 2024 May 21;16:1401109. doi: 10.3389/fnagi.2024.1401109 (PMC11148439; doi:10.3389/fnagi.2024.1401109)
Supplement: Supplementary file 3 [file Data_Sheet_1.DOCX]

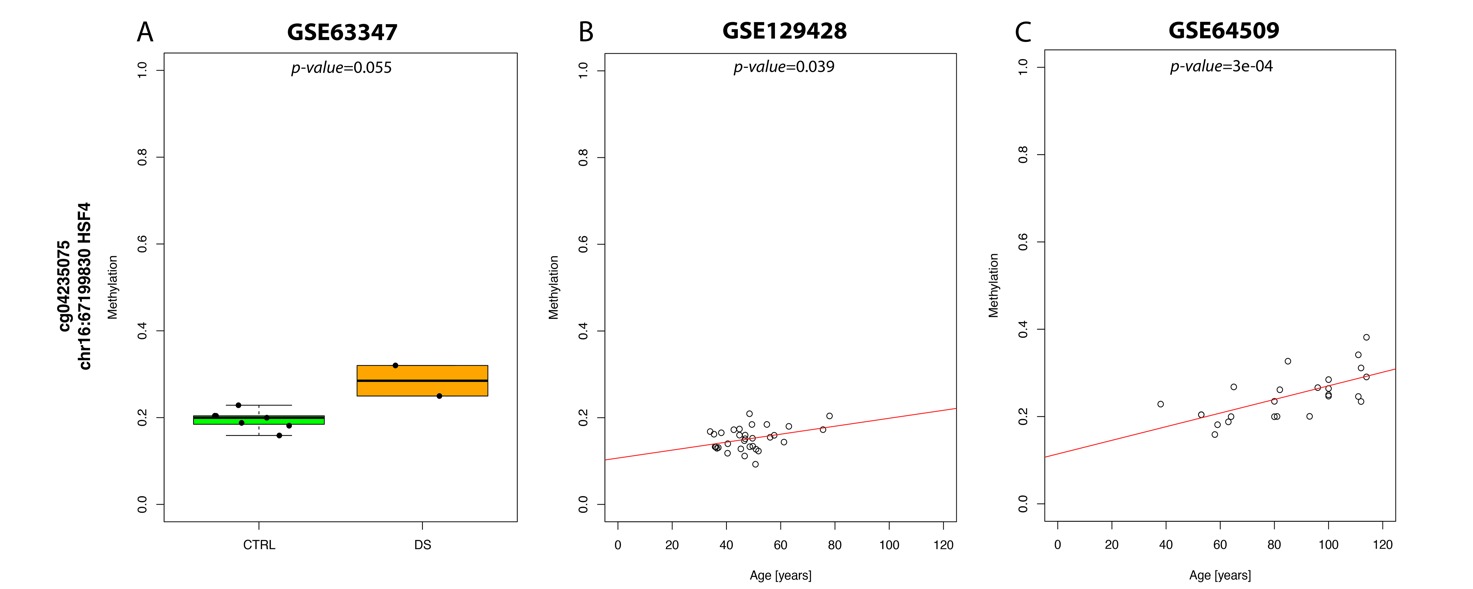


**Supplementary Figure 1. DNAm profiles of *HSF4* CpGs in human hippocampi.** A) DNAm profiles of Illumina Infinium 450k probe cg04235075 in hippocampus from subjects with DS and euploid controls (GSE63347) (nominal p-value calculated with Mann-Whitney test). B, C) DNAm profiles of Illumina Infinium 450k probe cg04235075 in hippocampus from subjects without overt pathologies at different ages (GSE129428, GSE64509) (nominal p-values calculated with linear model).
